# Assessment of the Influence of the Selected Range of Visible Light Radiation on the Durability of the Gel with Ascorbic Acid and Its Derivative

**DOI:** 10.3390/ijms23158759

**Published:** 2022-08-06

**Authors:** Iwona Golonka, Beata Kizior, Bartłomiej M. Szyja, Mateusz P. Damek, Witold Musiał

**Affiliations:** 1 Department of Physical Chemistry and Biophysics, Wroclaw Medical University, Borowska 211A, 50-556 Wrocław, Poland; 2 Faculty of Chemistry, Wrocław University of Science and Technology, ul. Gdańska 7/9, 50-344 Wrocław, Poland

**Keywords:** ascorbic acid, stability, degradation, ethyl ascorbic acid, UV radiation, photosensitivity, gels, substance-polymer interaction, DFT method

## Abstract

(1) Background: Depending on the type of hydrophilic polymer used, different types of hydrogels may be chemically stable or may degrade and eventually disintegrate, or dissolve upon exposure to sunlight. Many over-the-counter medications are now stored with a limited control of temperature, humidity and lighting. Therefore, in this study, the photostability of a gel made of cross-linked polyacrylic acid (PA), methylcellulose (MC) and aristoflex (AV) was assessed, and the interaction between the polymers used and ascorbic acid and its ethylated derivative was investigated. (2) Methods: The samples were continuously irradiated at constant temperature for six hours. The stability of the substance incorporated into the gels was assessed using a UV-Vis spectrophotometer. FTIR-ATR infrared spectroscopy was used to measure changes during the exposure. (3) Results: Ascorbic acid completely decomposed between the first and second hours of illumination in all samples. The exception is the preparation based on polyacrylic acid with glycerol, in which the decomposition of ascorbic acid slowed down significantly. After six hours of irradiation, the ethylated ascorbic acid derivative decomposed in about 5% for the polyacrylic acid-based gels and aristoflex, and in the methylcellulose gel it decomposed to about 2%. In the case of ascorbic acid, the most stable formulation was a gel based on polyacrylic acid and polyacrylic acid with glycerol, and in the case of the ethyl derivative, a gel based on methylcellulose. (4) Conclusions: The experiment showed significant differences in the decomposition rate of both compounds, resulting from their photostability and the polymer used in the hydrogel.

## 1. Introduction

Ultraviolet radiation is divided depending on the wavelength into three main areas that cause different biological effects and reach different layers of the skin: UV-C (100–280 nm), UV-B (280–315 nm) and UV-A (315–400 nm). It is important to note that radiation reaching the surface of the Earth varies with the solar altitude, which depends on the geographic location, season and the time of day [1]. This may influence the stability of pharmaceutical gels depending on the gel type with possible decomposition and dissolution of polymer components [2].

The beginning of the interest in gels dates back to 1960 [3]. Gels are polymers linked in a three-dimensional network that are able to absorb water or body fluids of a mass ranging from 10% to several thousand-folds of the dry weight of the polymer. Their biocompatibility potential guaranteed the polyacrylic acid derivatives and methylcellulose a permanent place in the pharmacopeia, i.e., for the topical forms of the drug. They are applicable in pharmaceutical technology and tissue engineering [4]. In an aqueous environment, polyacrylic acid reacts with atmospheric oxygen. The consequence of this process is the permanent decrease in or loss of gel viscosity. This process is catalyzed by sunlight, UV radiation and the presence of certain metals in the solution [5], including traces of iron ions and other transition metals. At a pH over 10, polyacrylic acid is not subject to aerobic degradation; therefore, gel alkalization can prevent the loss of its rheological properties during irradiation. The photodegradation inhibition can be obtained by water-soluble UV absorbers added to the gel, e.g., benzophenone-2 [6] or benzophenone-4 (CTFA). In many gel formulations, these absorbers are used in combination with disodium or tetrasodium salts of ethylene diamine tetra-acetic acid (EDTA) [7,8]. The gamma-irradiated PVA/MC blends possessed higher energy of activation when compared to that of unirradiated blends, depending on the stage of thermal decomposition. This is due to the higher crosslink density of the PVA component [9]. Ammonium acryloyldimethyltaurate/VP copolymer (AV) can be used in the preparation of gels. AV does not contain carbohydrates and aromatic solvents. It is used as a gelling substance in aqueous systems and a thickening agent in emulsions of “o/w”. The stabilizing effect of AV is explained by the structure of the polymer, which enables coating of droplets or solids particles, (e.g., pigments). AV is a copolymer, which because of its hydrophobic functionals is used as an effective combining agent. Due to the hydrophilic sulfonic groups, the polymer interacts with biologically active substances in formulations, and may prolong their activity on the skin surface [10]. AV was assessed as more stable in comparison to other derivatives of polyacrylic acid [11].

L-ascorbic acid (AA), or vitamin C, is a water-soluble antioxidant that has been used in personal care formulations and pharmaceutical preparations for many years [12]. Ascorbic acid participates in many key processes that take place in the human body. Unfortunately, people are not able to produce it on their own. It is essential to deliver it to the body with food. Topical or local routes can provide a source of ascorbic acid, which is demonstrated by the fact that local application promotes surgical healing and better tissue reconstruction, as the oral route has limitations in terms of providing high concentrations of ascorbic acid to the peripheral structures of the skin [13]. Antioxidants are necessary for neutralizing the radical oxidation species (ROS) formed in the skin by UV exposure [14]. AA is equally effective against both UVB and UVA radiation effects and sites treated with topical AA showed a significant reduction of erythema after skin exposure to the UVB radiation [15]. The ethylated derivative of ascorbic acid (3-O-ethyl ascorbic acid, AE) is a currently very intensively studied form of a derivative of vitamin C [16]. It was developed for enhancement of the stability of the molecule and the perseverance of the aqueous solubility. Unlike the other derivatives of ascorbic acid, it does not have to be first converted into ascorbic acid inside the skin, but is directly used by the skin, just like L-ascorbic acid, and most importantly it is currently the most stable form of vitamin C soluble in water [17,18].

The exposition of AA and AE to environmental factors, including UV-VIS radiation, may hinder the effective application of such systems, especially on the skin regions available to sunlight. The purpose of this work was to analyze the photostability of ascorbic acid and its derivative in a hydrophilic environment of polymeric hydrogels. It was also important to determine the substance-polymer interaction using UV-radiation, FTIR-ATR infrared, statistical methods and the computational method (DFT) to select the most stable formulation.

## 2. Results

### 2.1. Irradiated Substrates

In the initial experiment, the UVA and UVB spectra of the substrates, excluding AA and AE, were reordered every 1 h in a period of 6 h. During the whole irradiation process, no changes were observed in the spectra of evaluated samples.

#### 2.1.1. PA Gel with Ascorbic Acid and 3-O-ethyl-L-ascorbic Acid

**Figure 1:**
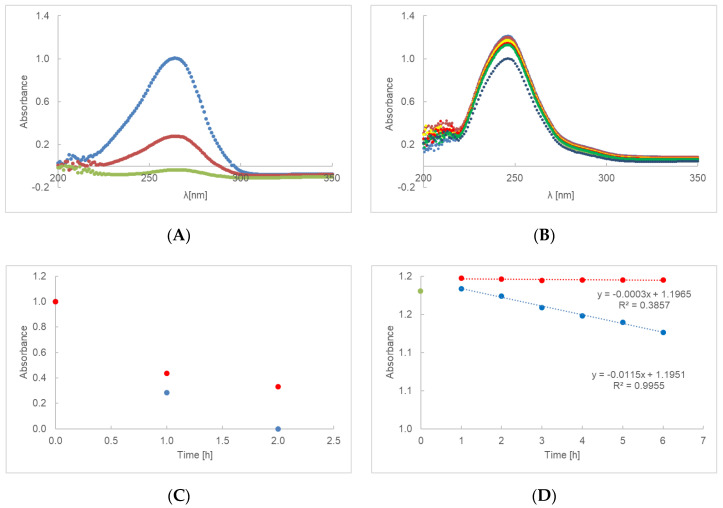
UV-Vis spectra of preparation AA-PA (**A**) and AE-PA (**B**), irradiated at 0(**–**), 1(**–**),2(**–**), 3(**–**), 4(**–**), 5(**–**) and 6(**–**) h. The UV degradation gel containing PA and ascorbic acid (**C**), or ethyl ascorbic acid (**D**), presented as time function of absorbance. Red dots represent unexposed samples and blue dots represent irradiated samples. During the first hour, the specimen stabilizes—green dot represents the starting point of the procedure on (**D**) graph.

The UV-Vis spectra of PA gel with ascorbic acid (AA-PA) and ethyl ascorbic acid (AE-PA) are shown in Figure 1A,B. The first measurement was made after the preparation of the gel sample and then after each subsequent hour of irradiation until the ascorbic acid signal disappeared at a wavelength of 266 and, in the case of ethylated ascorbic acid, at a wavelength of 247 nm. Decomposition of ascorbic acid is observed between the first and second hour of irradiation (Figure 1C). The non-irradiated gel AA-PA shows a slower decrease in the absorbance value of about 35% compared to the average absorbance measured for the five irradiated samples.

The degradation rate constant for the irradiated preparation AE-PA was 0.0115 h^−1^ and was close to the rate constant 0.0003 h^−1^ for the preparation containing AE-PA when not exposed to radiation (Figure 1D). The loss of ethylated ascorbic acid in AE-PA for the sample irradiated after 24 h was 15.7%.

#### 2.1.2. PA and Glycerol Gel with Ascorbic Acid and 3-O-ethyl-L-asdcorbic Acid

**Figure 2:**
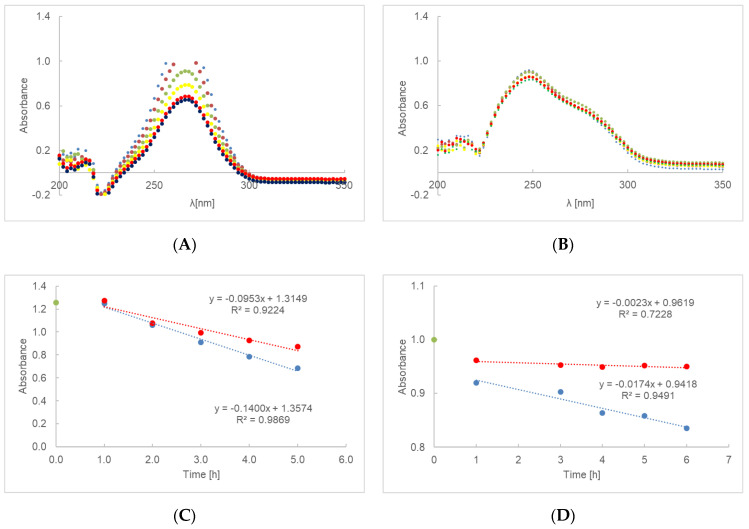
UV-Vis spectra of preparation AA-PA-G (**A**) and AE-PA-G (**B**), irradiated at 0(**–**), 1(**–**),2(**–**), 3(**–**), 4(**–**), 5(**–**) and 6(**–**) h. The UV degradation gel containing PA and ascorbic acid (**C**), or ethyl ascorbic acid (**D**), presented as time function of absorbance. Red dots represent unexposed samples and blue dots represent irradiated samples. During the first hour, the specimen stabilizes—the green dot represents the starting point of the procedure on (**D**) graph.

The UV-Vis spectra of AA-PA-G and AE-PA-G are shown in Figure 2A,B, respectively. In a gel based on polyacrylic acid and glycerin, we observed much slower decomposition of AA than on the basis of other polymers. Therefore, it was possible to determine the rate constant, which was 0.1400 h^−1^ for the irradiated AA-PA-G and 0.0953 h^−1^ for the non-irradiated AA-PA-G (Figure 2C).

The degradation rate constant for the irradiated AA-PA-G was 0.0174 h^−1^, whereas in the case of non-exposed AA-PA-G the rate was 0.0023 h^−1^ (Figure 2D).

#### 2.1.3. MC Gel with Ascorbic Acid and 3-O-ethyl-L-asdcorbic Acid

**Figure 3:**
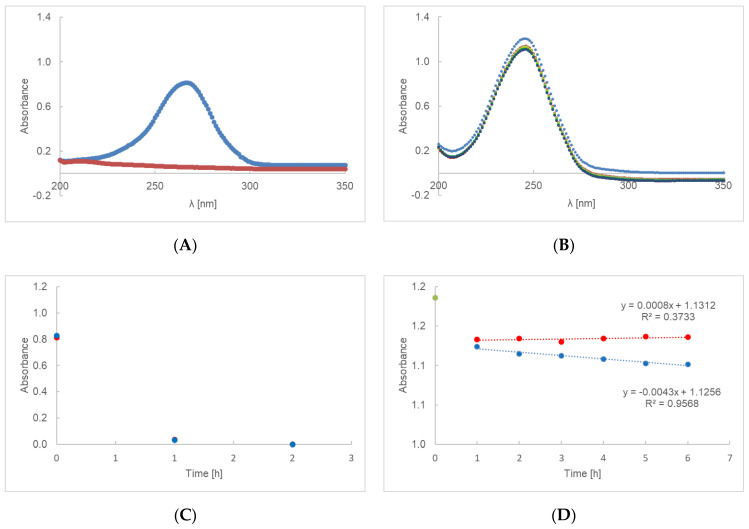
UV-Vis spectra of preparation AA-MC (**A**) and AE-MC (**B**), irradiated at 0(**–**), 1(**–**),2(**–**), 3(**–**), 4(**–**), 5(**–**) and 6(**–**) h. The UV degradation gel containing PA and ascorbic acid (**C**), or ethyl ascorbic acid (**D**), presented as time function of absorbance. Red dots represent unexposed samples and blue dots represent irradiated samples. During the first hour, the specimen stabilizes—green dot represents the starting point of the procedure on (**D**) graph.

The UV-Vis spectra of AA-MC or AE-MC are presented in Figure 3A,B. Comparing the irradiated and control samples, no differences in the rate of AA decomposition were observed (Figure 3C). The dependence of the absorbance of AE (Figure 3D) on time is described by a linear equation where the decomposition rate constant for the irradiated sample is 0.0043 h^−1^ and for the non-irradiated sample 0.0008 h^−1^. After 6 h of irradiation, the amount of AE decreased by 2%. The loss of ethylated ascorbic acid in preparation AE-MC for the sample irradiated after 24 h was 12.8%.

#### 2.1.4. AV Gel with Ascorbic Acid and 3-O-ethyl-L-asdcorbic Acid

**Figure 4:**
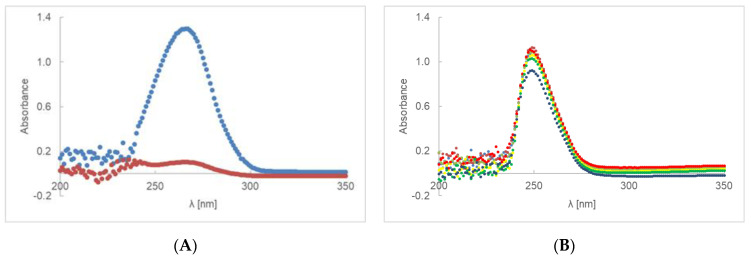

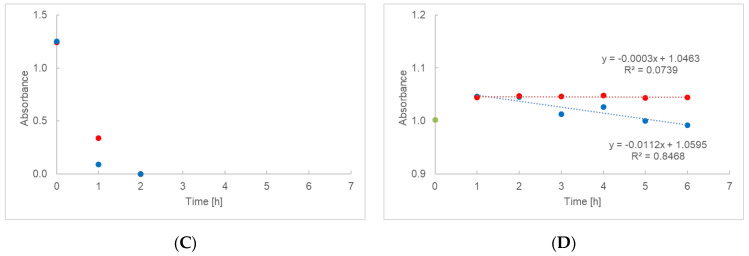
UV-Vis spectra of preparation AA-AV (**A**) and AE-AV (**B**), irradiated at 0(**–**), 1(**–**),2(**–**), 3(**–**), 4(**–**), 5(**–**) and 6(**–**) h. The UV degradation gel containing PA and ascorbic acid (**C**), or ethyl ascorbic acid (**D**), presented as time function of absorbance. Red dots represent unexposed samples and blue dots represent irradiated samples. During the first hour, the specimen stabilizes—green dot represents the starting point of the procedure on (**D**) graph.

The UV-Vis spectra of AA-AV or AE-AV are shown in Figure 4A,B. As for the previous samples, the first absorbance measurement was carried out after the preparation of gels containing ascorbic acid and then every hour. In the case of the irradiated sample, we observe the signal disappearance after the first hour (Figure 4A,C), which is not noticeable for the second preparation (Figure 4B).

The dependence of the absorbance of AE (Figure 4D) on time is described by a linear equation where the decomposition rate constant for the irradiated sample is 0.0112 h^−1^ and for the non-irradiated sample 0.0003 h^−1^. After 6 h of irradiation, the amount of AE decreased by 3%. The loss of ethylated ascorbic acid in the AE-AV preparation for the sample irradiated after 24 h was 10.9%.

### 2.2. Viscosity

**Figure 5:**
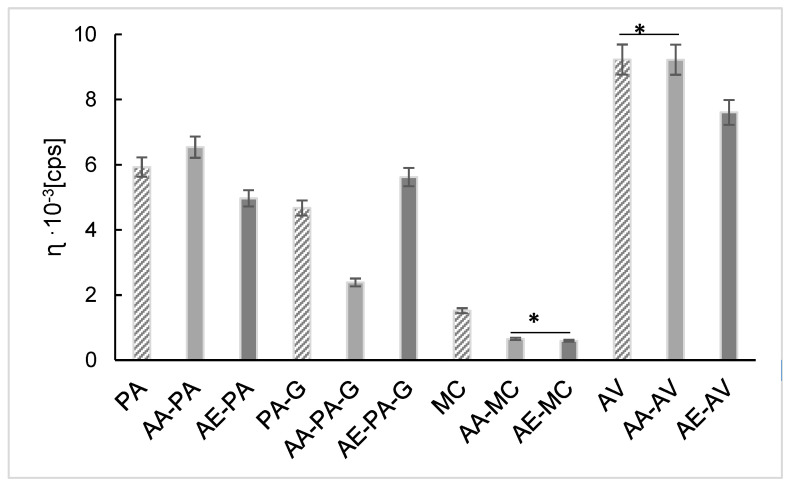
The viscosities of preparations AA–PA, AA–PA–G, AA–MC, AA–AV, AE–PA, AE–PA–G, AE–MC and AE–AV at a temperature of 20 °C. Asterisk (*) indicate no differences (*p* > 0.005, ANOVA test) between AA–MC and AE–MC and AV and AA–AV preparations.

The obtained results from the viscosity study of preparations AA-PA, AA-PA-G, AA-MC, AA-AV, AE-PA, AE-PA-G, AE-MC and AE-AV are presented in Figure 5. The lowest value of hydrogel viscosity was observed for gels based on methylcellulose and the highest for gels based on aristoflex. Statistical ANOVA analysis of the hydrogel viscosity showed differences between the most viscosities of the preparations, e.g., gels based on PA, PA-G, MC and AV polymers without the addition of ascorbic acid or its derivative. Differences are also observed when comparing the viscosity of gels with AA and gels with its ethylated derivative. It was found that there was no difference in viscosity between AA-MC and AE-MC and AV and AA-AV formulations as indicated by asterisks in Figure 5.

The addition of ascorbic acid increases the viscosity of the polyacrylic acid gel and the addition of the derivative reduces it. This phenomenon is the opposite for a polyacrylic acid-glycerin-based hydrogel. The obtained values of the discrepancies between the means are listed in Table 1.

### 2.3. Physicochemical Properties of Ascorbic Acid and 3-O-ethyl-L-ascorbic Acid

The basic physicochemical properties of ascorbic acid and its derivative calculated in ACD application are presented in Table 2. The data confirm the assumption on the important variabilities of the two acids.

### 2.4. Photostability Mechanism

We have carried out the theoretical investigations of the photostability of the ascorbic acid and its ethylated derivative in an attempt to explain the differences determined experimentally. First of all, we have observed the differences in the absorption spectra of the protonated and deprotonated (anionic) forms of ascorbic acid, which allowed us to form the following hypothesis: the differences in the excitation and the relaxation mechanisms of the neutral and anionic form of ascorbic acid indicate that the local environment can be responsible for the observed effect. In essence, the deprotonation reaction can easily occur depending on the local pH of the gel, which can lead to the different behavior.

**Figure 6:**
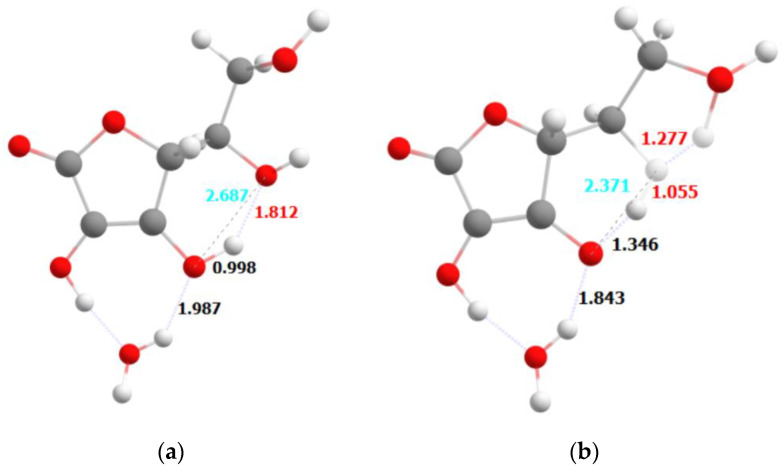
The geometries of the ascorbic acid in its ground state (**a**) and the conical intersection (**b**).

Figure 6 shows the optimized geometries of the neutral form of the ascorbic acid in its electronic ground state (Figure 6a). Absorption of a photon leads to the rearrangement of the hydrogens in the molecule. Protons are transferred from O1 to O2 atom (see Figure 6), and from O2 to the terminal hydroxyl group, what can be explained by the mechanism of electron-coupled proton transfer. The final structure contains a quasi-ring formed by the proton in between the O2 and terminal oxygen atom. This oxygen atom, together with the H atom from the hydroxyl group and one forming the quasi-ring (H2) assume the shape resembling H_2_O molecule. Thermal motion of the atoms make it possible that H_2_O would dissociate from the ascorbic acid, giving rise to the radical or carbocation species in the terminal position, what in turn might lead to the decomposition of this species.

The final structure is not a stationary point on the potential energy surface, but rather it is within the region of conical intersection. That means that upon excitation, the energy of a photon absorbed can be dissipated in a form of heat through atomic vibrations. An important observation is that the O1 atom loses the proton in the process, which resembles the deprotonation of the molecule in a low pH environment.

**Figure 7:**
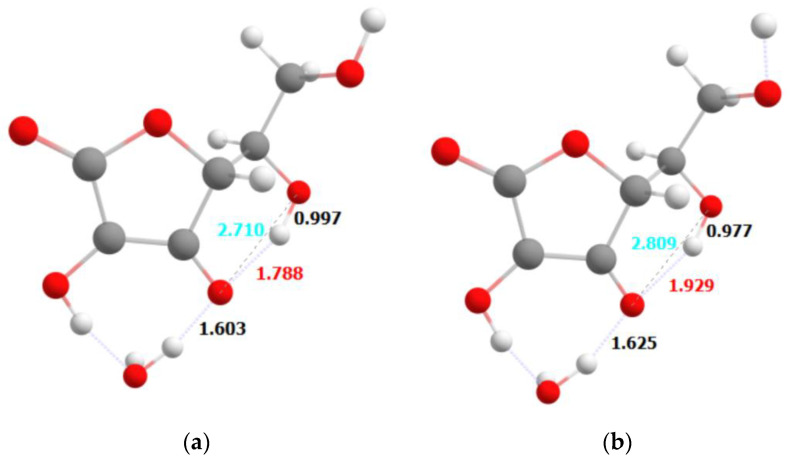
The optimized geometry of the deprotonated form of the ascorbic acid in its ground state (**a**) and first excited state (**b**).

**Figure 8:**
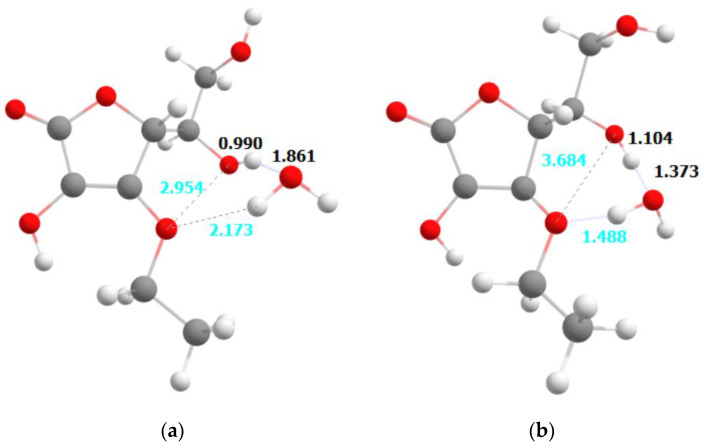
The optimized geometries of the ethylated form of the ascorbic acid in its ground electronic state (**a**) and the first excited singlet state (**b**).

Slightly different behavior has been observed for the deprotonated (anionic) form of the ascorbic acid. This form does not have the proton bound to the O1 atom, similarly to the proton transfer upon the excitation described above. The optimized geometries of the ground state and in the first excited state are shown in Figure 7 and Figure 8a,b, respectively.

The geometry in the ground state is characterized by a strong hydrogen bond between the O1 and H2 atoms, forming a quasi-ring. Upon excitation, the quasi-ring stays intact, but weakening of the O-H bond in the terminal hydroxyl group is observed. Similar effect has also been observed for the neutral form of the ascorbic acid.

The separation of the ground state from the first excited singlet state is only 0.5 eV. This also suggests that the geometry might be in the region of the conical intersection; however, keeping in mind the shortcomings of the TDDFT method, we should not consider it as a proof. On the other hand, the mechanism of the relaxation from the first excited singlet to the ground state will still be based on the thermal dissipation. It can also be noticed that the lack of the proton does not allow the second quasi-ring to be formed.

Significantly different observations have been made for the ethylated form of ascorbic acid. Upon excitation, the molecule does not significantly change its geometry—we do not observe any proton transfers or weakening of the bonds within the ascorbic acid molecule. The only significant change is the strengthening of the intermolecular hydrogen bond formed by the O1 atom and H_2_O molecule. This change, however, has a stabilizing character. The observation is consistent with the relatively large vertical difference between the ground and first excited state, amounting to 1.7 eV. That means the relaxation to the ground state should involve the emission of the photon in the near-infrared range.

### 2.5. FTIR Spectroscopic Studies

The FTIR spectra of pure powdered ascorbic acid and ethylated ascorbic acid were included in Supplementary Materials Figure S1A,B. Signals in the range 3524–3206 cm^−1^ in the spectrum of ascorbic acid corresponded to different hydroxyl groups [19,20]. The CH band stretching occurred at 3000, 2711 and 2916 cm^−1^. The characteristic absorption peak at 1752 cm^−1^ in the spectra of ascorbic acid was due to the stretching vibrations of the C=O of the five-membered lactone ring. The C=C stretching band appeared at 1651 cm^−1^. Various vibrational bands were observed in the region 1200–1500 cm^−1^ that were connected with the CH_2_ scissoring, twisting and wagging and the C-H deformation modes. At 1497 cm^−1^ and 1454 cm^−1^, there was CH bending, 1389 cm^−1^ and 1364 cm^−1^ CH_2_ wagging and C-O-H bending. Then, at 1271, we observed COC stretching, 1247 cm^−1^ COH bending (twisting), 1221 cm^−1^ and 1197 cm^−1^ CC (= O) -O stretching, 1111 cm^−1^ COC stretching, 1076 cm^−1^ and 1044 cm^−1^ COC stretching and COH bending, 1021 cm^−1^ CH bending, 870 cm^−1^ and 820 cm^−1^ CC ring stretching, 754 cm^−1^ and 720 cm^−1^ OH out-of-plane deformation and 680 cm^−1^ OH out-of-plane deformation/C-C ring stretching. The planar-ring deformation mode and the non-planar-ring deformation modes were correlated to our experimentally observed wavenumbers 680 cm^−1^ and 564 cm^−1^, respectively. The C=O out-of-plane bending mode was found to have calculated wavenumber 751 cm^−1^ with the observed wavenumber 722 cm^−1^ and, in these studies, 720 cm^−1^ [21]. In the case of the ascorbic acid derivative, we did not notice four signals coming from the OH group, only one at 3306 cm^−1^. The 1753 signal (C=O stretching) was shifted to 1739 cm^−1^ and 1651 cm^−1^ (C=C stretching) to 1671 cm^−1^. The signals from the C-H stretching of the methylene groups were more pronounced in AA than in AE. The FTIR spectra of the AA-PA, AA-PA-G, AA-MC, AA-AV, AE-PA, AE-PA-G, AE-MC and AE-AV preparations stored in the dark and irradiated were included in the Supplementary Materials. In Figures S2, S4 and S8 of preparations containing ascorbic acid, peaks in the range 3200–3520 cm^−1^ corresponded to the different hydroxyl groups [22]. This was not observed in the case of preparations containing glycerol (Figure S6) because these groups were obscured by the OH groups derived from glycerol [23]. The characteristic absorption peak at about 1750 cm^−1^ in the spectra of the preparations AA-PA, AA-MC and AA-AV resulted from the C=O stretching vibration of the five-membered lactone ring and did not disappear in the irradiated preparations. The signals at about 3030 cm^−1^ and 2917 cm^−1^ corresponded to CH stretching and decay in the case of the AA-AV irradiated preparation, producing a signal at 3057 cm^−1^. The signal around 1650 cm^−1^ was generated by the vibrations of the CC ring stretching. Various vibrational bands were observed in the region 1200–1550 cm^−1^ that were connected with the CH_2_ scissoring, twisting and wagging and the CH deformation modes. The band at about 1277 cm^−1^ was originated by COC stretching. In addition, very strong COC stretches were seen at 1111 and 1024 cm^−1^ in the IR spectrum. A strong signal at 757 cm^−1^ belonged to the OH out-of-plane deformation. In the AA-PA sample, we did not observe any differences during illumination. In AA-PA-G, the signal at 569 cm^−1^ shifted to 560 cm^−1^. In the case of AA-AV, the signal at 2916 cm^−1^ and 473 cm^−1^ disappeared during irradiation. However, the signals shifted in the area of the occurrence of the following groups: stretching C=O, stretching C=C, bending CH, stretching COC and bending COH. The characteristic changes observed in the preparations are presented in Table 3.

In preparations containing ethyl ascorbic acid (AE-PA, AE-PA-G, AE-MC and AE-AV), instead of a few strong signals from the OH group, as in ascorbic acid, there were weaker or only one occurring in the 3400–3200 range cm^−1^. At about 2931 cm^−1^, C-H stretching of the alkane group was found. In some spectra, there was a signal at about 2100 cm^−1^, possibly from the C-H group on the aromatic ring. In the AA-AV and AE-AV spectra (Figures S8 and S9), the signal from the S=O group stretching in the sulfonate was observed at approximately 1036 cm^−1^.

### 2.6. Statistics

For a first-order reaction, as shown in the Figure 1C,D, Figure 2C,D, Figure 3C,D and Figure 4C,D, the plot of the absorbance [A] versus time was a straight line, where the slope of the straight line corresponds to the negative rate constant k. The unit of the rate constant of a first-order reaction was h^−1^. Thus, a change in the concentration did not change the numerical value of k in a first-order reaction. The linear correlation parameters of preparations AE-PA, AA-PA-G, AE-PA-G, AE-MC and AE-AV, which were photo-protected and then exposed to radiation, were shown in Table 4. This table does not show the data for the gel preparations based on polymers of polyacrylic acid, methylcellulose and aristoflex containing ascorbic acid (AA-PA, AA-MC and AA-AV) because the decomposition of the active substance took place after about 2 h. The exception here was a gel based on polyacrylic acid with glycerol—preparation AA-PA-G. The lowest rate constants among the irradiated samples were observed for gels based on methylcellulose with ethyl ascorbic acid. The straight line for the irradiated gel based on PA and the gel based on AV showed a similar level of inclination. The coefficients for the straight lines were, respectively, −0.0115 and −0.0112. The straight line for irradiated methylcellulose-based gel had a slope factor of about 2.5 times lower, equal to −0.0043. Among the unexposed samples, the gels based on aristoflex showed the highest rate constants, while the lowest rate constants were observed for the gels based on polyacrilic acid. The correlation coefficient defining the level of linear dependence between the absorbance and the time of unexposed and irradiated samples is presented in Table 4. The results indicate that the correlation is positive, i.e., that an increase in the value of one feature was accompanied by an increase in the mean value of the second characteristic. In the case of the unexposed preparations AE-PA, AE-MC and AE-AV, the linear correlation coefficient was very low; that is, the linear relationship was not fulfilled and, thus, no changes in absorption were observed over time. The standard errors in estimating the coefficients a and b were less than 0.05. The same was true for the estimation error of y for all samples.

#### F-Test for Linear Correlation and Student’s *t*-Test

To verify whether the changes in absorbance of the samples under non-irradiation conditions for 6 h were statistically significant, the F-test was performed. The null hypothesis was that there were no statistically significant differences between the variables and the alternative hypothesis was that there were statistically significant differences between the variables. A confidence interval of 0.95 was adopted. A probability greater than 0.05 did not warrant rejecting the null hypothesis. The obtained results were presented in Table 5. The obtained probability values for all gels clearly exceed the value of 0.05, which did not warrant rejection of the null hypothesis. This means that there were no statistically significant differences between the non-irradiated samples. On the basis of the F-test, the stability of non-irradiated gel samples containing AE may be determined in the first 6 h of the experiment.

Student’s *t*-test was used to test for statistically significant differences between the exposed and unexposed samples. The calculated parameters are shown in Table 5. Hypothesis H_0_ implies that the radiation does not affect the sample. The alternative hypothesis H_1_ implies that the radiation affects the sample. With a confidence level of 5%, the *t*-test results rejected the null hypothesis and accepted the alternative hypothesis in all tested samples. In all cases, statistical differences of the behavior of ethyl ascorbic acid in the different gels could be confirmed depending on whether the sample was irradiated or not, as Table 4 and Table 5 do not include data for AA-PA, AA-MC and AA-AV formulations. In the case of these irradiated and non-irradiated samples, we observed complete decomposition of ascorbic acid after about 2 h.

Student’s *t*-test was used to test for statistically significant differences between the exposed and unexposed samples. The calculated parameters were shown in Table 5. Hypothesis H_0_ implies that the radiation did not affect the sample. The alternative hypothesis H_1_ implied that the radiation affects the sample. With a confidence level of 5%, the *t*-test results rejected the null hypothesis and accepted the alternative hypothesis in all tested samples. In all cases, statistical differences of the behavior of ethyl ascorbic acid in the different gels could be confirmed depending on whether the sample was irradiated or not, as Table 4 and Table 5 do not include data for AA-PA, AA-MC and AA-AV formulations. In the case of these irradiated and non-irradiated samples, we observed complete decomposition of ascorbic acid after about 2 h.

## 3. Discussion

The rapid degradation of AA poses challenges to local delivery of the molecule in pharmaceutical and cosmetic products. It is dry-stable, but unstable in solution, especially in alkaline solution. It easily oxidizes under the influence of air [24]. On the basis of UV-Vis spectra of AA-PA, AA-MC and AA-AV preparations, the disappearance of absorbance at the maximum absorption of the AA molecule was observed after about 2 h in irradiated and non-irradiated samples. The physiological activity of L-ascorbic acid stems from its basic functional structure. It is a five-membered lactone sugar acid and its C3 and C2-enolic hydroxyl groups. In solution, the delocalization of π electrons in the molecule over the 2,3-enediol moiety conjugated with the C1 carbonyl group, make the proton on the C3 hydroxyl group significantly more acidic and to dissociate with a pKa of 4.25. Dissociation of the second hydroxyl takes place at pKa of 11.57 [22]. The C2 and C3-OH groups are critical in electron donation and therefore in oxidation of AA [25]. Preparations containing the AE molecule, where the signal from the ascorbic acid derivative was visible even after 24 h in the preparations subjected to irradiation, proved to be much more stable. Structural modification of the compound AE protected the 3-OH group from ionization, and thus the molecule from oxidation, but also results in changes in the physicochemical properties. EA was found to have a pKa of 7.72 at room temperature [26]. Comparing the pKa of compounds AA and AE it appears that the ascorbic acid derivative was a weaker acid, which may have resulted in its higher stability. The addition of glycerol in the case of the AA-PA-G formulation slowed the fading out of the signal at the AA absorption maximum. Looking at previously published literature data, glycerol may affect the acid–base balance, and our calculations showed that the protonated and deprotonated form of AA exhibit a different photostability mechanism [27]. Glycerol does not absorb in UV and will have no direct effect on the photochemistry of the system. On the other hand, its presence may affect the behavior of substances such as quercetin in the gel, which has already been observed [28]. Bandelin and Tuschhoff observed the effects of ethanol, glycerin, propylene glycol, sorbitol, sucrose, corn sugar and dextrose on the breakdown of ascorbic acid much earlier [29]. There is an interaction between the solute and solvent molecules in a solution, and the number of these interactions increases as the number of solute molecules is greater. Glycerin–water mixtures have been well-tested experimentally and theoretically from various angles such as molecular structure, dynamics and the hydrogen bond network. For anhydrous glycerol, only the glycerol–glycerol interaction should be considered, and for glycerol–water mixtures, the glycerol–glycerol and glycerol–water interaction should be considered. It is known that in in the liquid phase, the three OH groups of the glycerol molecule can be involved in about six hydrogen bonds with of adjacent molecules, while the water molecule can establish four hydrogen bonds; however, water–water interactions were observed in the literature data even at higher glycerol concentrations because of fluctuations in density [30,31,32,33]. The Plackett–Burman experimental design was used to investigate the compatibility of ascorbic acid with various syrup excipients. It was found that glycerin (5% *v*/*v*) had a significant stabilizing effect on ascorbic acid, while sugar cane syrup (33.3% *v*/*v*) had a destabilizing effect [34,35]. The carbonyl group is an electron acceptor. Thus, this group can has an electron withdrawing effect on ring, and consequently on the hydroxyl group, in ascorbic acid. In this condition, weaker hydrogen bonds are formed between solute and solvent molecules. The positive deviation can be due to generated ions when ascorbic acid dissolves in water (aqueous solution). It is well-known that the interaction between ions and water molecules is stronger than the interaction between neutral molecules and water molecules [36]. On the basis of the collected literature, it appears that the slower decomposition of ascorbic acid in the AA-PA-G preparation compared to the AA-PA preparation may be caused by the formation of hydrogen bonds, all the more so as efforts to elucidate the action of glycerol should focus not only on the local structure of the water, but on the extended network of hydrogen bonds in the system. It is also known that the solubility of ascorbic acid is much lower in glycerol and is 0.01 g per 1 mL and in water 0.33 g per 1 mL, which is used in pharmaceutical formulations [37]. Certainly, glycerol does not have antioxidant properties [38]. Hydrogen bonds play a key role in the dissolution of organic substances in water and other solvents and thus participate in biological and physical processes [39]. Consequently, propanotriol can undergo a large number and variety of chemical transformations such as selective oxidation, dehydration and hydrogenolysis, and esterification, but in the presence of catalysts, enzymes or microorganisms [40].

The preparations based on AV showed the highest viscosity and the lowest based on MC. When comparing the AA-PA and AA-PA-G preparations, the addition of glycerol decreased the viscosity, and the opposite was true in the preparations containing AE.

Theoretical studies of the photostability of ascorbic acid and its derivative are confirmed by experimental studies. Observed differences in the absorption spectra of the protonated and deprotonated (anionic) AA forms, indicating that the deprotonation reaction can easily take place depending on the local pH of the gel. Photon absorption causes the transfer of electron-coupled protons. The thermal movement of the atoms allows the dissociation of the H_2_O with ascorbic acid, resulting in the formation of a radical or carbocation at the end position, which in turn can lead to decomposition of this compound. In summary, it is important how many water molecules (protons) there are around an AA molecule. On the other hand, the AE molecule does not significantly change its geometry—we do not observe any proton transfers or weakening of bonds in the ascorbic acid molecule. The only significant change is the strengthening of the intermolecular hydrogen bond formed by the O1 atom and the H_2_O molecule. However, this change is of a stabilizing nature. The lack of changes in the bonding within the ascorbic acid upon excitation is consistent with the experimental observation of significantly better photostability of the ethylated form of ascorbic acid. The opposite observation is made for the neutral and anionic forms of ascorbic acid, where a significant rearrangement of the hydrogen atoms within the molecule is also consistent with lesser stability of these forms. This also explains the relatively small changes observed for the ethylated form mixed with the different gels—polyacrylic acid, methylcellulose and aristoflex. The local pH of the environment provided by these gels is not able to significantly alter the properties of the ethylated form of ascorbic acid. Contrary to that, depending on the gel, a local environment is able to affect the equilibrium between neutral and anionic forms of ascorbic acid, and lead to easier or more difficult decomposition of these species.

In the FTIR spectrum (Figure S3), the effect of irradiation causes the appearance of the signal at 780 cm^−^^1^ corresponding to C-H bending in 1,2,3-trisubstituted and differences in the range of vibrations outside the plane, e.g., at 569 cm^−^^1^ could be assigned to the out-of-plane C-O deformation modes of structure (Figures S4 and S5). These results are consistent with the data obtained from the calculation methods that radiation does not break down the molecules, but affects its absorption and thus the molecular dynamics. Upon irradiation of the AA-MC preparation, the FTIR spectra showed scissor signals in the CCO group, present in the monomer and the AA dimer. The spectra of unexposed and irradiated AE-MC samples (Figure S7) differ over the entire range. In spectrum (A) the signal was probably visible from the stretching group C=O in the lactone, which is not observed in spectrum (B). AA-AV gels show shifting signals in the spectra during irradiation and they lose scissoring signals in the CCO group. After AE-AV irradiation, signals from the C=C groups present in alkenes and lactones were observed in the FTIR spectrum (Figure S9B). Vibrations at approximately 1700 cm^−1^ belonging to the -COOH group with less intensity than signals in the range 1650 to 1500 cm^−1^ attributed to COO- stretching may suggest that there was an interaction between the AA carboxylate group (Figures S2, S6 and S8) and the PA carboxyl group. In most FTIR spectra, the AA-PA preparation is an exception here, during irradiation changes occur, which should be taken into account when making pharmaceutical or cosmetic preparations. Shifts in the wavelength of the occurrence of the signal from the CH2 stretching, swinging COH groups, and the stretching of the CO groups in CHOH may suggest interactions between the outer CH2 and the inner CHOH of the surrounding polyols [41].

The F-test indicated that there were no statistically significant differences between the non-irradiated samples for the given formulation. When using Student’s, statistically significant differences in the behavior of ethylascorbic acid in various gels and in the AA-PA-G preparation depending on whether the material was irradiated or not were repeated. The analysis was not performed for the preparations AA-PA, AA-MC and AA-AV as there was complete decomposition of ascorbic acid after about 2 h in irradiated and non-irradiated samples.

## 4. Materials and Methods

### 4.1. Materials

The following reagents were used in the experimental part: ascorbic acid (Pharma Cosmetic, Kraków, Poland), 3-O-ethyl ascorbic acid (Corum, Taipei, Taiwan), methylcellulose (MC) (CAS 9004-67-5, Sigma-Aldrich, Saint Louis, MO, USA), polyacrylic acid (abbreviation is used-PA, Carbomer, CARBOPOL^®^ 980 NF POLYMER, CAS 9007-20-9, Lubrizol, Wickliffe, OH, USA), sodium hydroxide (CAS 1310-73-2, Sigma-Aldrich, Saint Louis, MO, USA), Aristoflex AVC (abbreviation is used-AS, Clariant, Frankfurt am Main, Germany) and glycerol (CAS 56-81-5, Sigma-Aldrich, Saint Louis, MO, USA).

### 4.2. Preparation of Gels

In brief, the gel 0.3% *w*/*w* was prepared by the following procedure: polyacrylic acid resin (weight in grams) was dispersed in distilled water (volume according to the desired concentration of gel). The mixture was stirred until thickening occurred and then neutralized by drop-wise addition of 2.5 mmol NaOH, until a transparent gel appeared. The quantity of NaOH is adjusted to achieve gel with desired pH. In the case of a gel based on polyacrylic acid PA-G, additional glycerol was added in appropriate proportions. A 2% gel containing methylcellulose in its composition was obtained by mixing the appropriate amount of methylcellulose with water at a suitable temperature. A 2% gel containing AV in its composition was obtained by mixing the appropriate amount of AV with water at a suitable temperature. All gels were stabilized depending on temperature for 24 h. Preparation of AA-PA, AA-PA-G, AA-MC and AA-AV containing additional ascorbic acid consisted in adding to 4 g of the prepared gel 1 mL 7.0·10^−3^% ascorbic acid aqueous solution, whose absorbance is about one. Preparation of AE-PA, AE-PA-G, AE -MC and AE-AV containing additional ascorbic acid consisted in adding to 4 g of the prepared gel 1 mL 2.50·10^−3^% 3-O-ethyl ascorbic aqueous solution, whose absorbance is about one (Table 6).

### 4.3. Methods

#### 4.3.1. Spectrophotometric Method

The calibration curve and stability of preparations AA-PA, AA-PA-G, AA-MC, AA-AV, AE-PA, AE-PA-G, AE-MC and AE-AV was evaluated with a PG Instruments UV–Vis T60 spectrophotometer (Alab, Warszawa, Poland), interfaced with a computer. Samples were measured in quartz cuvette of path length 1 cm at 25 °C, using a total volume of 3 mL, applied in the range 190–900 nm. The samples were evaluated every 1 h from the initial stage, when the components were combined. The reported absorbance represents the average of five samples.

#### 4.3.2. Calibration Curve

The components included in the gel did not absorb in the measured range 210–440 nm. In addition, the measured absorbance of ascorbic acid and 3-O-ethyl ascorbic acid in all samples did not exceed 1.5, especially important for gels whose dilution would be difficult and would introduce additional errors. The relationship between absorbance and analyses concentration in conditions when the tested system meets the Lambert-Beer law is rectilinear and can be used to determine the concentration of analyses in the sample.

Calibration curves, graphical representation of the relationship between absorbance and concentration of standard substance were determined for the gel with the ascorbic acid and 3-O-ethyl ascorbic acid (AA-PA, AA-PA-G, AA-MC, AA-AV, AE-PA, AE-PA-G, AE-MC and AE-AV). They are described by equation y = ax + b. On their basis, the coefficients a and b of the linear function describing the equation of the standard curve and parameter errors were determined (Table 7). The coefficient of determination is determined by the R^2^ formula. It is a normalized value and takes values from zero to one.

#### 4.3.3. The UV Irradiation

The influence of solar radiation on the stability of ascorbic acid and its derivative in gels was investigated using a climatic chamber (KBF P 720, Binder, Tuttlingen, Germany) at 25 °C and 65% humidity. Lamps emitting radiation with a wavelength in the range of 320–800 nm were used in accordance with the ICH guidelines. The samples were placed in quartz cuvettes covered with polymer stoppers transmitting to UV-VIS radiation. The absorbance was measured at one-hour intervals for the first 6 h, and then after 24 h at a wavelength of 247 nm for ethylated ascorbic acid and 266 nm for ascorbic acid. The amount of degraded ascorbic acid and its ethylated derivative was calculated using the standard curves for each of the gels.

#### 4.3.4. Viscosity

The viscosity of the hydrogels was measured at the temperature of 20 °C by employing a rotational viscometer (Brookfield DV2T, Middleboro, MA, USA). The rotation speed of the spindle was fixed at 200 rpm. Standard spindles were used, respectively, No 06 for PA, PA-G and AV gel, the gels themselves and that with ascorbic acid or its derivative, No 04 and 3 for MC gel. The measurement parameters such as the rotation speed and spindle number were chosen to obtain scale coverage between 60% and 90%. Each measurement was performed five times to calculate the average value of the viscosity of the hydrogel (η) together with the standard deviation (SD).

#### 4.3.5. Physicochemical Properties of Ascorbic Acid and 3-O-ethyl-L-ascorbic Acid

The basic physicochemical properties of the compounds were calculated on the basis of their structure using the ACD/ChemSketch program.

#### 4.3.6. Density Functional Theory (DFT)

**Figure 9:**
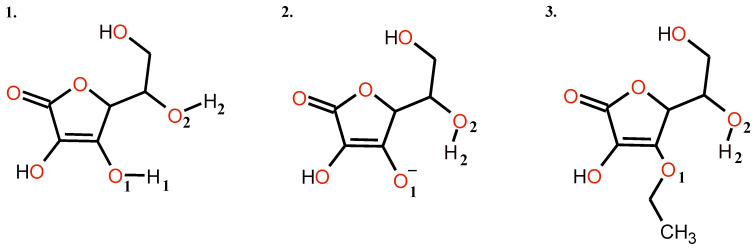
Structures of the studied forms of ascorbic acid: 1. neutral; 2. deprotonated; 3. ethylated.

All calculations in this study have been carried out within the framework of the DFT methodology, using Gaussian 16 Rev. C.01 suite of programs [42]. The ascorbic acid (Vitamin C) models are shown in Figure 9.

**Figure 10:**
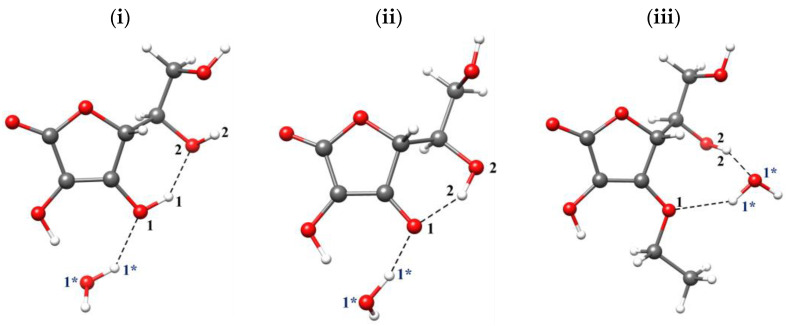
Optimized structures of microsolvation models of ascorbic acid in protonated (**i**), deprotonated (**ii**) and ethylated (**iii**) form. Carbon, oxygen and hydrogen atoms are shown in grey, red and white, respectively.

The PBE [43,44,45] functional and the def2-TZVP [46,47] basis set were applied. The solvent effect was accounted for by means of the polarizable continuum model (PCM) [48] with water used as a solvent. A single solvent molecule was added next to the investigated molecule to account for a local solvent environment in line with the microsolvation method (see Figure 9 and Figure 10). The geometries of all structures have been fully optimized, and the vibrational frequency calculations have been carried out to confirm that the obtained molecular structures corresponded to the minima on the potential energy surface (PES).

The electronic excitations have been calculated using the time-dependent DFT (TDDFT) method. To maintain consistency, the same functional and basis set combination was used. It has to be noted that TDDFT is known for an imperfect match between the calculated and experimental absorption spectra; however, typically, this is only quantitative rather than qualitative issue. The analysis was focused on the excitation and relaxation mechanisms for the investigated compounds. The energies of the first five singlets and five triplets have been determined, followed by the optimization of the S1 (first excited singlet state). The crossing between S0 (ground state) and S1 states—the conical intersection—was assumed when the last converged configuration along the optimization process showed a small difference between the ground and first excited state (below 0.1 eV).

#### 4.3.7. FTIR Spectroscopic Studies

FTIR measurements were performed using a Thermo Scientific Nicolet iS50 FT-IR Spectrometer with an attenuated total reflectance (ATR) device (Thermo Fisher Scientific, Waltham, MA, USA). Measurements were made for solid samples, gels and gels that were lyophilized using a 1-4 LD Alpha freeze dryer from Martin Christ GmbH (Osterode, Germany).

#### 4.3.8. Statistical Analysis

The experimental data were evaluated with the Statistica 10.0 software (Kraków, Poland), using the student’s *t*-test and F-test for linear correlation. The kinetic degradation rate constants were evaluated as a function y = A_1_ + A_0_e^−kt^ estimated with the use of the Gauss–Newton algorithm. The variability of viscosity preparations was analyzed employing ANOVA combined with Tukey’s honest significant difference test (Tukey’s HSD test).

## 5. Conclusions

Data on photostability, among other data indicating stability, are crucial for the development of pharmaceutical formulations; hence, industrial requirements for complete stability studies for most pharmaceuticals and drug substances are a priority. Our research shows that the presence of glycerol slows down the degradation of AA not only in room conditions but during irradiation, which has not been studied before. On the other hand, theoretical calculations show that photon absorption causes the transfer of electronically coupled protons and not the decomposition of the compound. The lack of binding changes in ascorbic acid after excitation is consistent with experimental observations of a much better photostability of the ethyl form of ascorbic acid. The FTIR-ATR spectra of pure AE and preparations containing it are also new. Summarizing theoretical, statistical and experimental calculations show that the addition of glycerol stabilizes AA and AE can be used in cosmetic products as a stable alternative to ascorbic acid, even on sunny days.

## Figures and Tables

**Table 1:** The differences between means obtained from Tukey’s test; the value of HSD was 0.188.

	AA-PA	AE-PA	PA-G	AA-PA-G	AE-PA-G	MC	AA-MC	AE-MC	AV	AA-AV	AE-AV
PA	0.6113	0.961	1.257	3.543	0.309	4.410	5.276	5.337	3.300	3.294	1.678
AA-PA		1.573	1.869	4.154	0.920	5.021	5.888	5.948	2.689	2.683	1.067
AE-PA			0.296	2.582	0.653	3.449	4.315	4.376	4.261	4.255	2.639
PA-G				2.286	0.949	3.153	4.019	4.080	4.557	4.551	2.935
AA-PA-G					3.234	0.867	1.734	1.794	6.843	6.837	5.221
AE-PA-G						4.968	4.968	5.028	3.609	3.603	1.987
MC							0.866	0.927	7.710	7.704	6.088
AA-MC								0.061	8.576	8.570	6.954
AE-MC									8.637	8.631	7.015
AV										0.006	1.622
AA-AV											1.616

**Table 2:** Comparison of selected physicochemical properties of ascorbic acid and 3-O-ethyl-l-ascorbic acid.

Naming	Ascorbic Acid	3-O-ethyl-L-ascorbic Acid
Molecular Formula	C_6_H_8_O_6_	C_8_H_12_O_6_
Molar Refractivity	35.26 ± 0.3 cm^3^	44.65 ± 0.4 cm^3^
Molar Volume	90.1 ± 3.0 cm^3^	139.0 ± 5.0 cm^3^
Polarizability	13.97 ± 0.5 10^−24^ cm^3^	17.70 ± 0.5 10^−24^ cm^3^
RDBE (Ring Double Bond Equivalents)	3	3
Average Mass	176.1241 Da	204.1773 Da
logP	−2.41 ± 0.45	−1.81 ± 0.52

**Table 3:** The specific and altered signals of preparation.

Preparation	Bands Alteration Regions Observed Unexposed [cm^−1^]	Bands Alteration Regions Observed after Irradiation [cm^−1^]
AA-PA	no differences	no differences
AA-PA-G	3278, 2935,2879, 1653,1564, 569, **480**	3274, 2934, 2880, **2066**, 1652, 1561, 560
AA-MC	1652, 1316, 1273, 1023	1656, 1318, 1274, 1024, **497**, **485**
AA-AV	3524, 3309, 3205, **3032**, **2916**, 1754, 1648, **1313**, **1274**, 1179, **1138**, 1024, 870, **473**, 447	3523, 3307, 3203, **3057**, 1755, 1644, **1299**, **1178**, 1151, 1036, 445
AE-PA	3305, 1673, 526	3313, 1671, **780**, 541
AE-PA-G	3278, 2936, 2879, **2144**, 1652, 1561, 1406, 1323, 1209, 1108, 1035, 994, 923, 851, **569**, **487**	3277, 2935, 2880, **1980**, 1654, 1565, 1408, 1325, 1208, 1110, 1040, 995, 924, 852, 560
AE-MC	3413, **3305**, **1744**, 1685, 1403, **1378**, 1330, **1200**, 1051, **945**, **885**, **821**, **761**, 460	**3466**, 3418, **2917**, **2850**, **2466**, 1660, **1440**, 1338, **1236**, 1061, **552**, 467
AE-AV	**3231**, **2886**, 2112, 1754, **1655**, 1548, 1443, **1420**, 1378, 1150, 1113, 1036, 937, 869, 761, 722, **507**	**3208**, **3070**, **2931**, **2100**, 1743, **1676**, **1647**, 1549, 1442, 1380, **1321**, **1293**, 1151, 1116, **1082**, 1035, 938, 872, 757, 732, **523**, **479**

New signals are written in bold.

**Table 4:** Linear correlation parameters for the obtained data.

Parameter	AE-PA		AA-PA-G		AE-PA-G		AE-MC		AE-AV	
	Irradiated	Unexposed	Irradiated	Unexposed	Irradiated	Unexposed	Irradiated	Unexposed	Irradiated	Unexposed
slope factor a	−0.0115	−0.0003	−0.1400	−0.0953	−0.0174	−0.0023	0.0043	0.0008	−0.0112	0.0003
coefficient b	1.1951	1.1965	1.3574	1.3149	0.9418	0.9619	1.1256	1.1312	1.0595	1.0463
standard error a	0.0004	0.0002	0.0093	0.0160	0.0020	0.0008	0.0005	0.0005	0.0024	0.0031
standard error b	0.0015	0.0008	0.0309	0.0529	0.0078	0.0034	0.0018	0.0020	0.0093	0.0120
linear correlation coefficient	0.9955	0.3857	0.9869	0.9224	0.9491	0.7228	0.9568	0.3733	0.8468	0.0653
standard error of y stimation	0.0016	0.0009	0.0294	0.0505	0.0084	0.0031	0.0019	0.0022	0.0100	0.0129

**Table 5:** Parameters of F-test and Student’s *t*-test.

*Parameters of F-test*					
Parameter	AE-PA	AA-PA-G	AE-PA-G	AE-MC	AE-AV
F	2.3830	47.543	64.405	0.3191	0.3192
*p*	*p* > 0.05	*p* > 0.05	*p* > 0.05	*p* > 0.05	*p* > 0.05
*Parameters of Student’s t-test*					
Parameter	AE-PA	AA-PA-G	AE-PA-G	AE-MC	AE-AV
*t*	25.1859	0.2210	5.7483	7.3800	0.6131
*p*	0.0039	0.0039	0.0039	0.0039	0.0039

**Table 6:** Composition of evaluated polymeric gels.

Samples Group	Sample Acronym	PA [g]	MC [g]	AV [g]	NaOH [g]	G-Glycerol [g]	Aqua [g]	AA [g]	AE [g]
**Basic gels**	PA	0.3	-	-	q.s. *	-	94.7	-	-
	PA-G	0.3	-	-	q.s. *	25	74.7	-	-
	MC	-	2	-	-	-	98.0	-	-
	AV	-	-	2	-	-	98.0	-	-
**Gels with** **AA**	AA-PA	0.3	-	-	q.s. *	-	94.7	0.035	-
	AA-PA-G	0.3	-	-	q.s. *	25	74.7	0.035	-
	AA-MC	-	2	-	-	-	98.0	0.035	-
	AA-AV	-	-	2	-	-	98.0	0.035	-
**Gels with** **AE**	AE-PA	0.3	-	-	q.s. *	-	94.7	-	0.013
	AE-PA-G	0.3	-	-	q.s. *	25	74.7	-	0.013
	AE-MC	-	2	-	-	-	98.0	-	0.013
	AE-AV	-	-	2	-	-	98.0	-	0.013

* 2.5 mmol of NaOH solution was added to the polymer dispersion.

**Table 7:** Linear correlation parameters for the standard curve y = ax + b.

Preparation	AA-PA	AA-PA-G	AA-MC	AA-AV	AE-PA	AE-PA-G	AE-MC	AE-AV
slope factor a	802.86	769.66	721.43	868.60	2008.4	402.909	2241.19	2036.7
standard error for a	37.53	50.12	59.80	49.64	80.47	65.786	81.500	112.45
coefficient b	−0.093	0.069	0.016	−0.017	0.054	−0.189	0.093	0.018
standard error for b	0.033	0.078	0.053	0.046	0.021	0.209	0.023	0.031
linear correlation coefficient	0.989	0.992	0.967	0.987	0.994	0.949	0.996	0.991

Preparation AA-PA, AA-PA-G, AA-MC, AA-AV, AE-PA, AE-PA-G, AE-MC and AE-AV concentration is expressed in *w*/*w*%.

## Data Availability

The data that support the findings of this study are available from the corresponding author upon reasonable request.

## Supplementary Materials

The following supporting information can be downloaded at: https://www.mdpi.com/article/10.3390/ijms23158759/s1.
