# Supplementary material for: Assessment of the Influence of the Selected Range of Visible Light Radiation on the Durability of the Gel with Ascorbic Acid and Its Derivative
Source: Int J Mol Sci. 2022 Aug 6;23(15):8759. doi: 10.3390/ijms23158759 (PMC9369315; doi:10.3390/ijms23158759)
Supplement: Supplementary file 1 [file ijms-23-08759-s001.zip › ijms-1827190-supplementary.pdf]

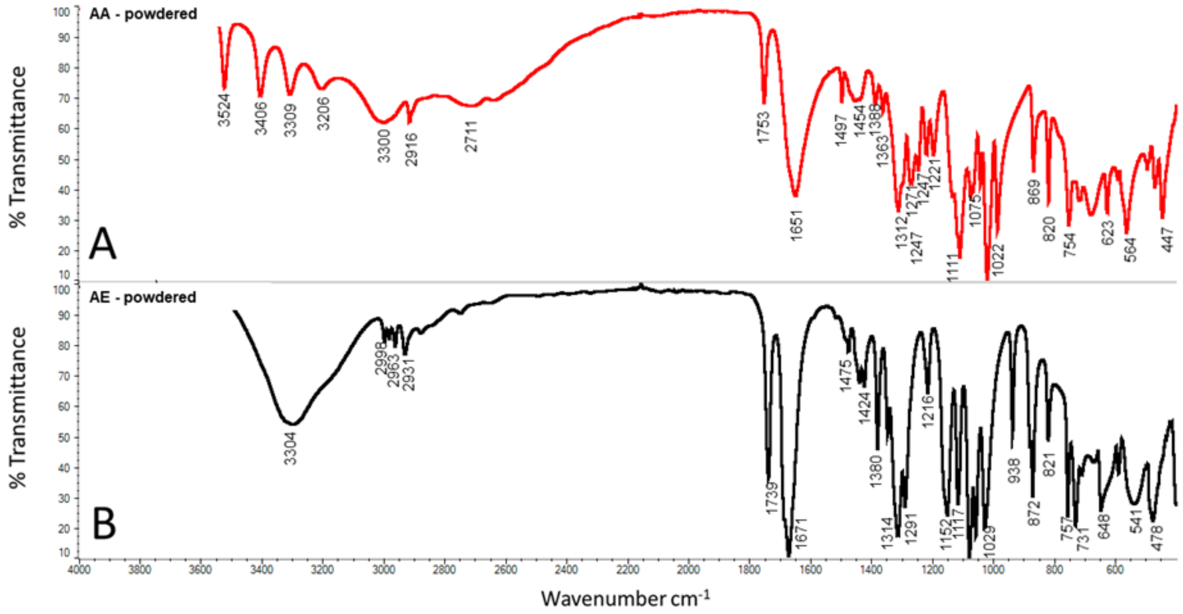

Figure S1. FTIR spectrum of dried preparation: (A) AA - powdered, (B) AE – powdered.

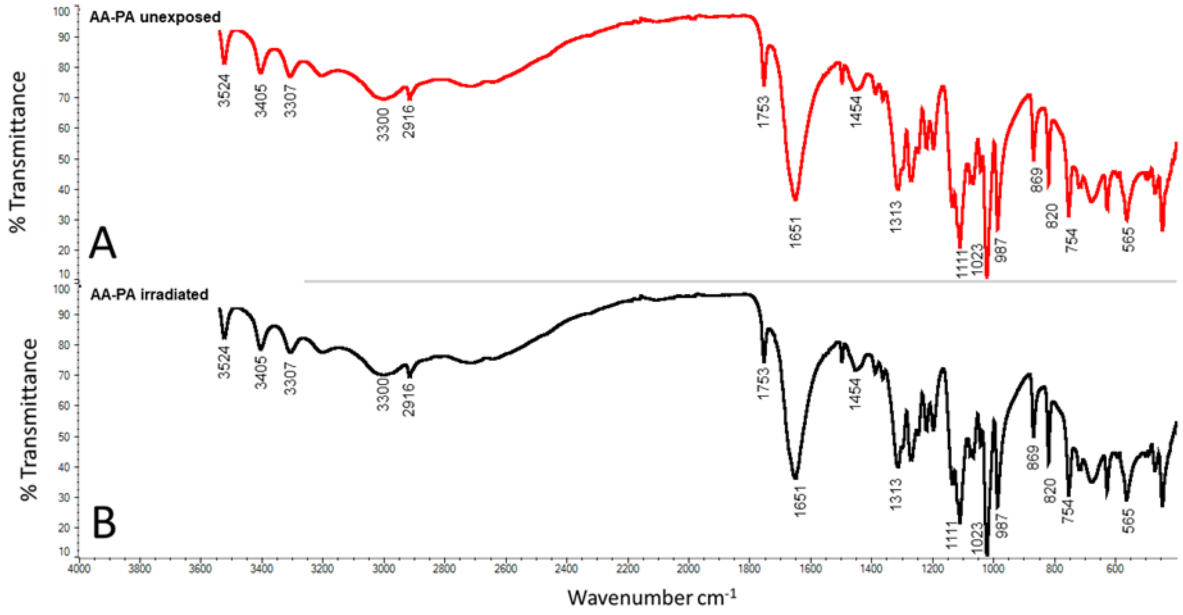

Figure S2. FTIR spectrum of freeze-dried preparation: (A) unexposed preparation AA-PA, (B) irradiated preparation AA-PA; composition of the preparation is given in the Table 2.

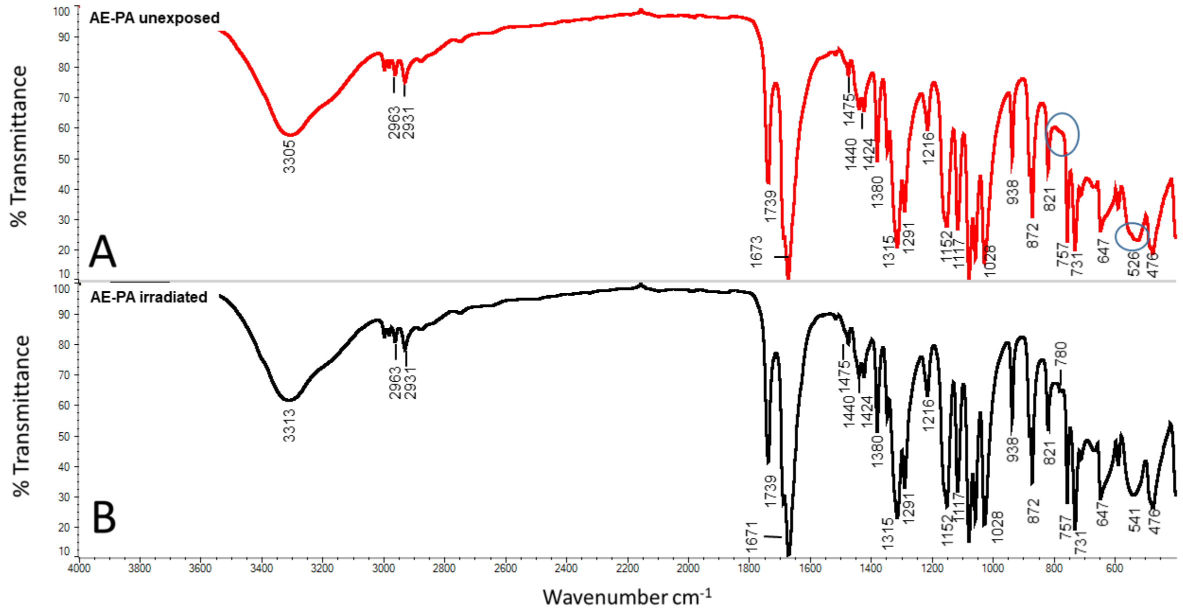

Figure S3. FTIR spectrum of freeze-dried preparation: (A) unexposed preparation AE-PA, (B) irradiated preparation AE-PA; composition of the preparation is given in the Table 2. The blue circle shows the differences.

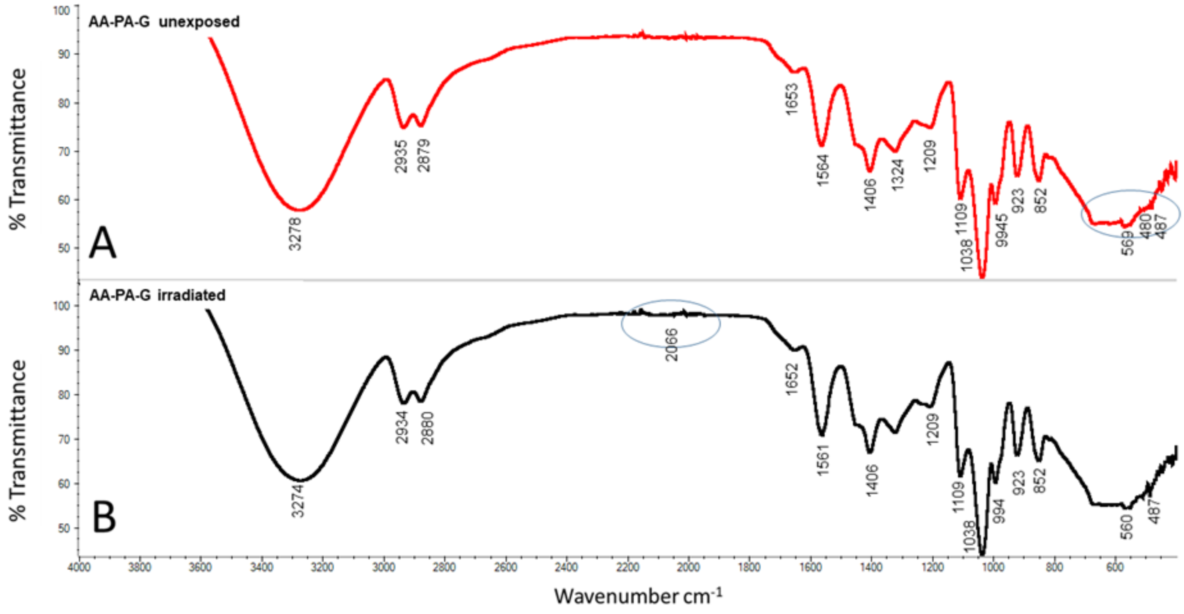

Figure S4. FTIR spectrum of freeze-dried preparation: (A) unexposed preparation AA-PA-G, (B) irradiated preparation AA-PA-G; composition of the preparation is given in the Table 2. The blue circle shows the differences.

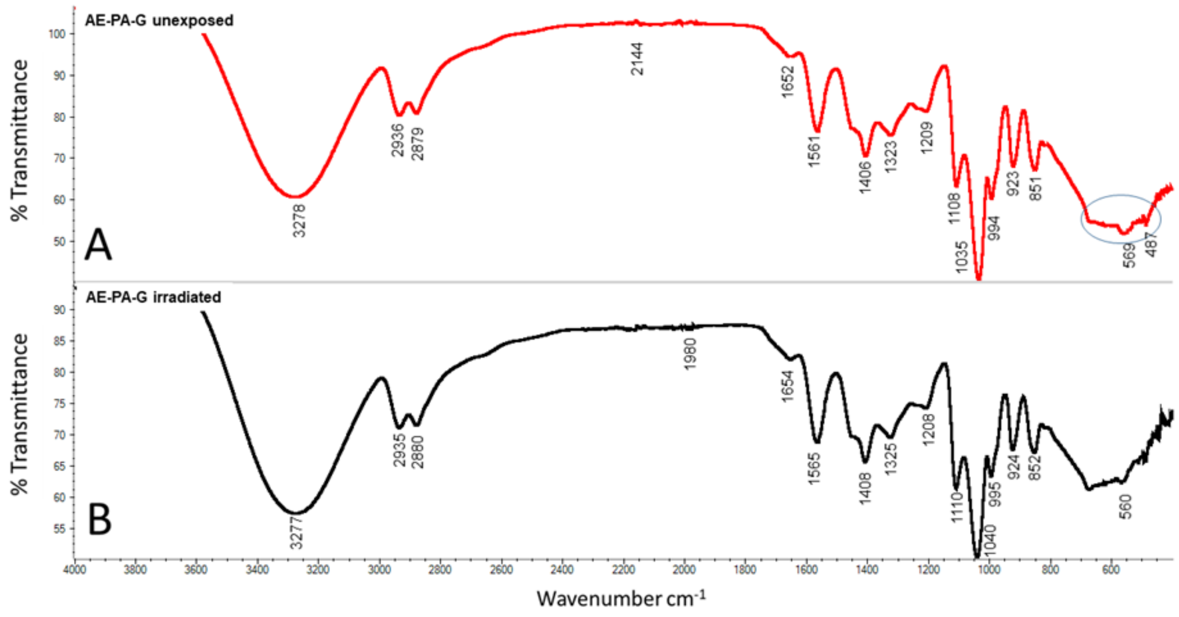

Figure S5. FTIR spectrum of freeze-dried preparation: (A) unexposed preparation AE-PA-G, (B) irradiated preparation AE-PA-G; composition of the preparation is given in the Table 2. The blue circle shows the differences.

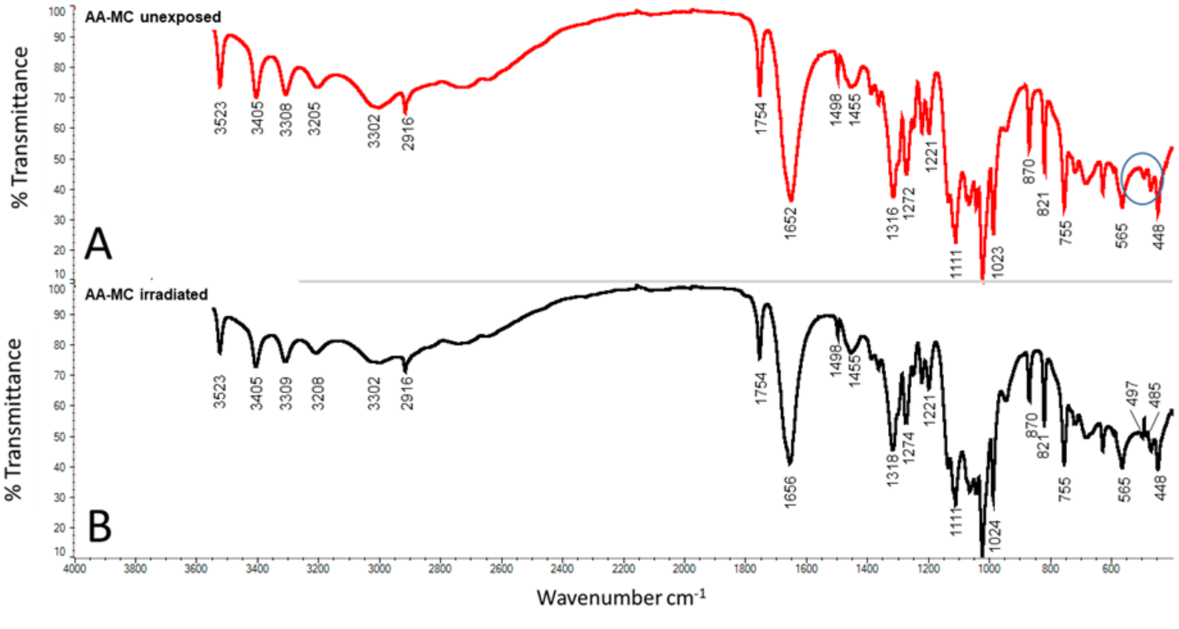

Figure S6. FTIR spectrum of freeze-dried preparation: (A) unexposed preparation AAE-MC, (B) irradiated preparation AA-MC; composition of the preparation is given in the Table 2. The blue circle shows the differences.

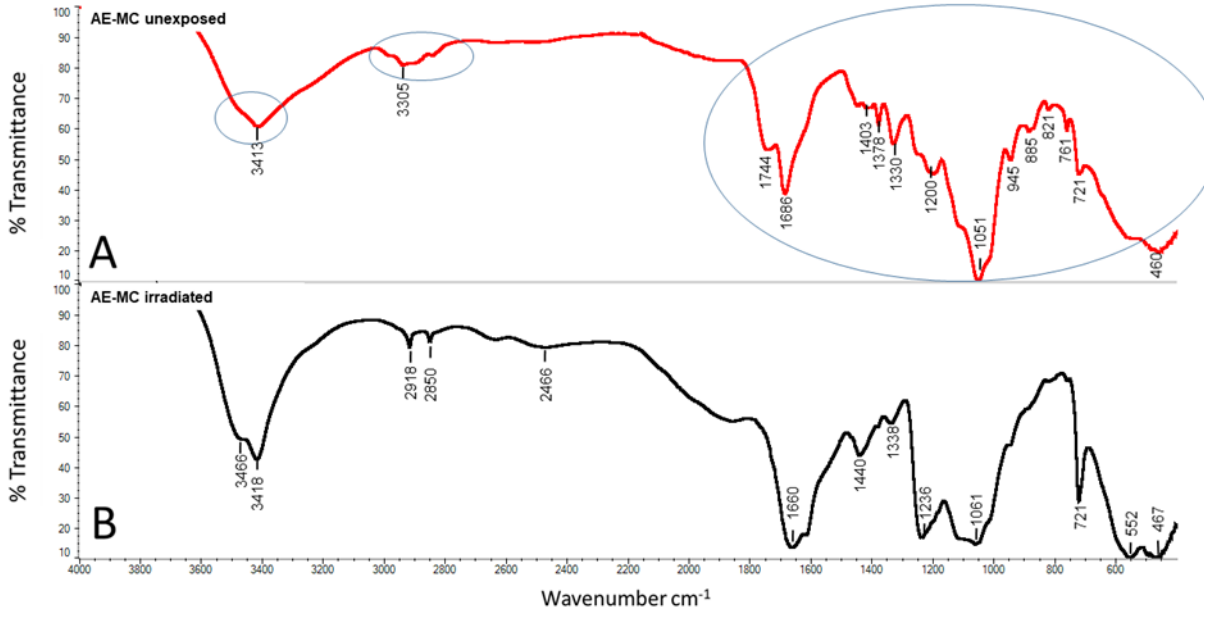

Figure S7. FTIR spectrum of freeze-dried preparation: (A) unexposed preparation AE-MC, (B) irradiated preparation AE-MC; composition of the preparation is given in the Table 2. The blue circle shows the differences.

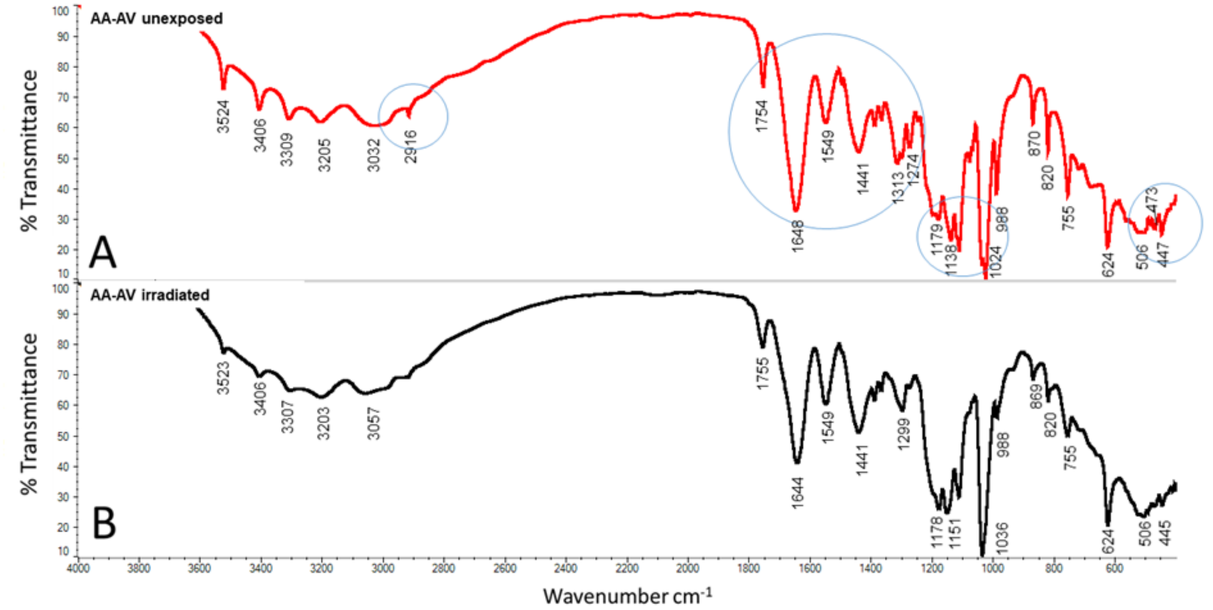

Figure S8. FTIR spectrum of freeze-dried preparation: (A) unexposed preparation AA-AV, (B) irradiated preparation AA-AV; composition of the preparation is given in the Table 2. The blue circle shows the differences.

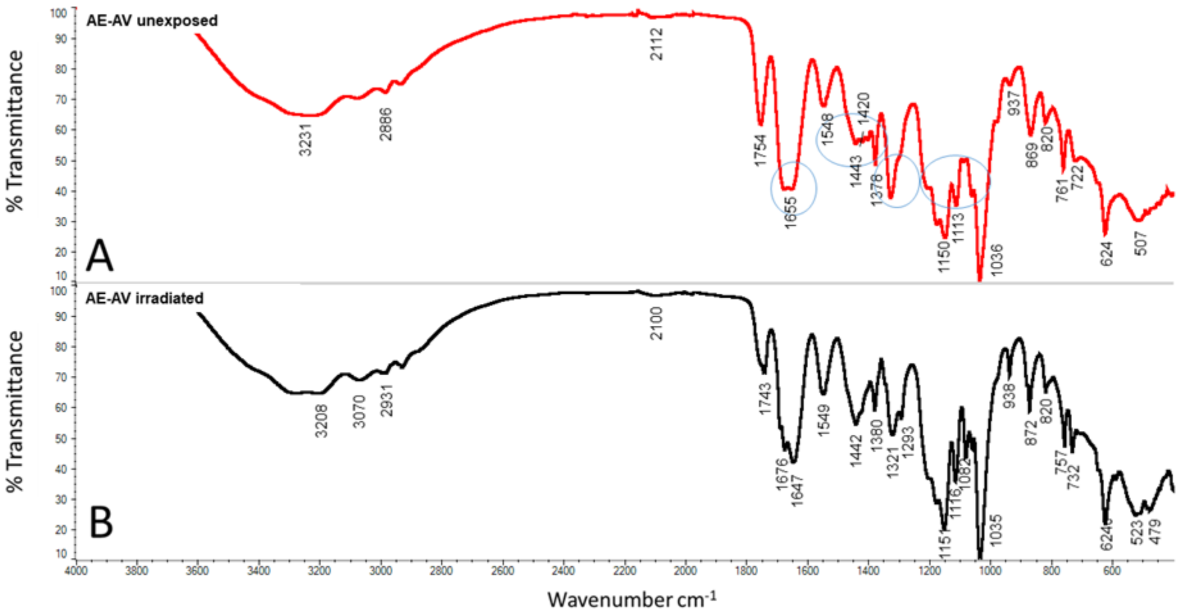

Figure S9. FTIR spectrum of freeze-dried preparation: (A) unexposed preparation AE-AV, (B) irradiated preparation AE-AV; composition of the preparation is given in the Table 2. The blue circle shows the differences.
